# Innovative Banana Fiber Nonwoven Reinforced Polymer Composites: Pre- and Post-Treatment Effects on Physical and Mechanical Properties

**DOI:** 10.3390/polym13213744

**Published:** 2021-10-29

**Authors:** K. Z. M. Abdul Motaleb, Abdul Ahad, Ginta Laureckiene, Rimvydas Milasius

**Affiliations:** 1 Faculty of Mechanical Engineering and Design, Kaunas University of Technology, 51424 Kaunas, Lithuania; ginta.laureckiene@ktu.lt (G.L.); rimvydas.milasius@ktu.lt (R.M.); 2 Department of Textile Engineering, BGMEA University of Fashion and Technology, Dhaka 1230, Bangladesh; abdulahadallsheikh@gmail.com

**Keywords:** banana, nonwoven, epoxy, polyester, eco-friendly composites, alkali treatment, water repellent treatment, gamma radiation

## Abstract

Four types of nonwovens were prepared from different sections of the banana tree e.g., outer bark (OB), middle bark (MB), inner bark (IB) and midrib of leaf (MR) by wet laid web formation. They were reinforced with two different types of matrices e.g., epoxy and polyester, to make eight variants of composites. Treatments including alkali on raw fibers, water repellent on nonwovens and gamma radiation on composites were applied in order to investigate their effects on properties of the composites such as water absorbency, tensile strength (TS), flexural strength (FS) and elongation at break (Eb%). Variations in the morphological structure and chemical composition of both raw banana fibers and fibers reinforced by the treatments were analyzed by Fourier Transform Infrared (FTIR) and Scanning Electron Microscopy (SEM). OB composites exhibited higher water absorbency, TS and FS and lower Eb% compared to other types of composites. Epoxy composites were found to have 16% lower water absorbency, 41.2% higher TS and 39.1% higher FS than polyester composites on an average. Water absorbency of the composites was reduced 32% by the alkali treatment and a further 63% by water repellent treatment. TS and FS of the composites were on average improved 71% and 87% by alkali treatment and a further 30% and 35% by gamma radiation respectively.

## 1. Introduction

Throughout the last century, traditional materials like wood, metal, ceramics, and glass have been rapidly replaced by polymer matrix composite (PMC) materials with reinforced synthetic fibers thanks to their many advantages, which include light weight, easy processing, low cost, and high productivity. However, this rapid increase of non-biodegradable PMC has also created dangerous and alarming problems such as environmental pollution from plastics, burning of fossil fuels, increased global warming potential, and more, which can create a harmful and unsafe environment for humans, animals, and marine life [1]. For these reasons, researchers are paying attention to alternative eco-sustainable, renewable, and degradable materials [2]. Natural fiber reinforcement, also called as green filler, can be a potential alternative to polymer composites due to numerous advantages including biodegradability, low relative density, inexpensiveness, ease of handling, ready availability, light weight, high impact resistance, high flexibility, low specific gravity, recyclability, low carbon emissions, good thermal and acoustic insulation, and more [3,4,5,6,7,8]. Natural fiber-reinforced composites (NFRCs) are becoming more attractive in many areas of engineering application, with a wide range of properties [9]. Natural fibers used as reinforcement in NFRCs can be obtained from plants, animals and minerals. However, most of the natural fibers investigated as reinforcement to date have been from agricultural plant byproducts or waste. Moreover, the recent advancements in biomaterials are not only limited to the extraction of natural fibers, but also include biobased matrices from, for instance, natural camphoric acid, epoxidized soybean oil [10], wasted cottonseed protein [2], natural deep eutectic aqueous solution of *Acanthopanax senticosus* stems [11], olive leaf waste [12] and more.

There are numerous sources of plant fibers all over the world, especially in tropical regions. Banana fiber is one of them, extracted from the stem of the banana tree *(Musa acuminata).* The stem, usually known as the pseudostem, is cylindrical in shape and contains plenty of long fibers. Banana fibers consist of 71.08% cellulose, 12.61% hemicellulose and 7.67% lignin in their chemical composition, with a diameter of 138 µm and density of 1.28 g/cm^3^ [13]. In a tropical country such as Bangladesh, banana plants are considered agricultural crops, growing abundantly due to favorable climate conditions. After harvesting the fruits, banana plants are cut at the lower section and the whole cutting portions are considered a complete waste; including pseudostem and leaf, these can be utilized as a source of natural fibers for the manufacturing of NFRCs, textiles, nonwovens, packaging materials, wiping materials, etc. [14]. The fibers can be used in industry without any additional expense in terms of cultivation [15]. Moreover, banana fibers exhibit good mechanical properties when compared with other cellulosic fibers, which makes them a potential reinforcing material in a variety of engineering applications [9,16,17,18].

However, the performance of the NFRCs depends on fiber orientation, amount, length, shape, and their interfacial bonding with the matrix [19]. Fiber orientations are also varied, with different forms of reinforcement including chopped fiber reinforcement, continuous fiber (filament) reinforcement, woven fabric reinforcement and nonwoven reinforcement. Woven fabrics are generally produced by interlacing the yarns, usually at the right angles, by following a regular pattern. The strength of woven fabrics can be increased by increasing the twist angle of the yarns up to a certain limit. However, this twist angle plays an opposite role in the case of composites. Increasing the twist angle decreases the permeability of the matrix to the fiber, which results poor fiber–matrix adhesion and low mechanical properties [20]. In the case of filament reinforcement, mechanical properties are much lower in the transverse direction of fibers, which is also a limitation for different applications. To avoid these problems, nonwoven reinforcement can be a great option. Nonwovens are prepared in a flat structure with different thickness, without interloping or interlacing. Fibers are chopped, uniformly distributed, and bonded together by chemical, mechanical or thermal treatment. Nonwovens have no preferential strength direction and can be produced on a large scale due to their ready availability and low cost [21].

Thermosetting resins are the resins most widely used as a polymer matrix in the composite industry. Among these, epoxy and polyester are the resins most typically applied as a matrix. Epoxy resins, also known as poly-epoxides, have good adhesion properties with natural fiber. Other key features include low moisture absorption, high chemical resistance, low shrinkage, and simple processing. These excellent properties make them a superior alternative with a wide range of applications [22]. However, unsaturated polyester resins, also known as polyhydric alcohols, have satisfactory mechanical properties and sufficient adhesion properties when used with the natural fibers, as reported in several studies. The main advantage of polyester resins is that they are cheaper, easily available, and can be used in a wide range of applications [23].

There is no doubt that natural fibers have a lot of potential due to their unique properties and environmental friendliness. However, they do have some drawbacks as well, such as high moisture absorption, low compatibility with the commercial resins, poor adhesion between fiber and matrix, less homogenous filaments, and low resistance to fire [17]. However, these challenges can be overcome through different types of physical and chemical treatments. Chemical treatment of the fibers, including alkaline, saline, acetylation, benzoylation and many more, can improve adhesion between fiber and matrix [24]. Alkali treatment is one of the simplest and least expensive methods; it is easily applicable to natural fibers by immersing them in a solution of NaOH. Following alkali treatment, the fibers become more uniform thanks to the removal of impurities. Consequently, the physical and mechanical properties of composites can be improved [17]. Several studies have been reported the improvement of physical and mechanical properties of composites through alkali treatment of natural fibers [25,26,27,28,29,30]. To overcome the problem of high water absorbency in NFRCs, surface treatment of natural fibers with water repellent is a potential solution. The water repellent makes a coating on the fiber surface and prevents water from penetrating inside. We could find no relevant studies to date investigating water repellent treatment of natural fibers to improve the hydrophobicity of NFRCs.

Physical treatments such as X-ray, ultraviolet (UV) ray, gamma ray, plasma, and corona can be applied to improve fiber matrix adhesion. Due to its low time consumption, high productivity, low environmental pollution, structural availability, and easy application, gamma radiation treatment is becoming more popular [31]. Gamma radiation is a powerful ionizing radiation which can penetrate inside the polymeric structure of composites and produce reactive sites, leading to a more well-oriented polymeric structure and thus improving the mechanical properties of the composite [32]. Many researchers have applied this radiation and reported improvement in the mechanical properties of the composites [32,33,34,35]. However, the studies also reported that gamma radiation improves the mechanical properties up to a certain level of gamma radiation dose, after which that it alters the properties. Therefore, an optimal dose must be maintained.

Numerous studies have been noted regarding the properties of banana fiber reinforced composite materials. The majority of them used banana fiber or pseudostem mats as a reinforcing material [15,16,18,36,37]. Only a few of them studied banana in nonwoven reinforced composite materials. Kenned J, et al. studied the thermo-mechanical and morphological characterization of needle punched banana fiber nonwoven reinforced polymer composites [13]. The properties of needle punched nonwoven from banana fiber were also studied by Sengupta et al. [38]. Thilagavathi et. al., developed needle punched banana nonwovens for application as noise control in car interiors [39]. However, no research has been reported on the development of banana fiber nonwoven using the wet laid web formation technique from various parts of banana tree, or on the properties of the resulting reinforced composites. Moreover, no study has been found regarding the surface modification of such nonwovens and their composites.

The aim of this study is to develop an ecofriendly composite material from a natural source which will be used in light weight applications in different areas, including packaging, household furniture, building materials, technical textiles and many more, with the potential to replace the existing environmentally destructive and carcinogenic synthetic composite materials that are still mostly being used today. To fulfil this objectives, four types of banana fiber nonwovens were developed from different parts of the banana tree: the outer bark, middle bark, inner bark of the stem, and mid rib of the leaf, using the wet laid web formation technique with the extracted fibers. The four prepared types of nonwoven were reinforced on two different types of matrices, epoxy resin and polyester resin, to make eight different composites. Surface treatments were applied in three stages: (i) in the fiber stage, alkali (NaOH) treatment to improve mechanical properties and hydrophobicity; (ii) in the nonwoven stage, water repellent treatment to improve hydrophobicity; and (iii) in the composite stage, gamma radiation treatment to improve the mechanical properties. The morphological structure and chemical composition of the raw fibers and treated fibers were each analyzed by FTIR spectroscopy and SEM micrography. The water absorbency of the composite samples was inspected, as was the improvement of hydrophobicity via surface modification. Mechanical properties including tensile and flexural strength and the influence of the physical and chemical treatments on those properties were also analyzed. A comparative study between the composites made using epoxy matrix and those which used polyester matrix is elaborated in different aspects throughout the study.

## 2. Materials and Methods

### 2.1. Materials

Completely matured and healthy banana trees at the age of 13 months were collected as waste material from an agriculture farm in Gazipur, Bangladesh after the fruits has been harvested). The cultivar of was “Amrit Sagar” of the species *Musa acuminata.* Epoxy resin, hardener HY-951, polyester resin and methylethylketone peroxide (MEKP) were bought from Nord Composites, Condé-Folie, France Caustic soda and water repellent (Nuva^®^ N2114 liq.) were purchased from Archroma International Ltd., Mölndal, Sweden. All the chemical materials used in this study were laboratory grade with high purity.

### 2.2. Methods

#### 2.2.1. Banana Fiber Extraction

**Figure 1:**
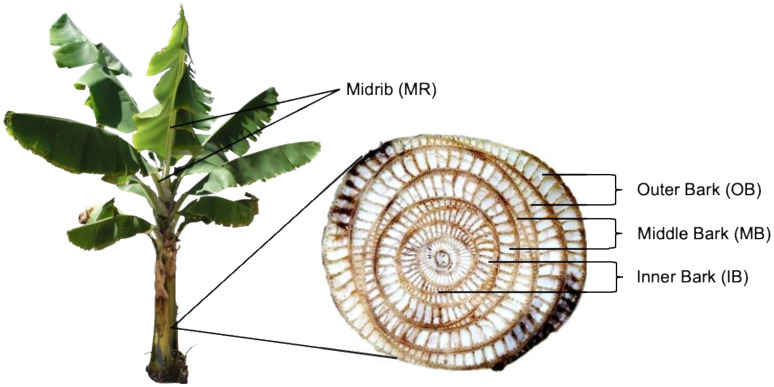
Banana tree and cross-section of banana stem.

Banana trees were segregated into four different sections: (1) the outer layers of banana bark, designated as outer bark (OB); (2) the middle layers of banana bark, designated as middle bark (MB); (3) the inner layers of banana bark, designated as inner bark (IB); and (4) the middle rib of banana leaves, designated as midrib (MR). All of these sections are shown in Figure 1. The raw materials from each section were then pressed by a metal tube squeezer to remove the inside water as much as possible. Afterwards, they were dried in sunlight for about 15 days.

The dried materials were scratched by a metal comber to make ribbons and then cut to a length of 3 cm. For the initial fiber extraction, these small pieces were placed in a large metal pot and treated with 5% (*w*/*v*) NaOH with a temperature of 90 °C for about 30 min until they became soft. They were rinsed thoroughly to removing unwanted materials and subsequently dried. In this way the raw banana fibers from different parts of the banana trees were extracted.

#### 2.2.2. Alkali Treatment of Fibers

The extracted raw fibers still contained various impurities such as fat, wax, pectens and so on. To remove these impurities, the fibers were immersed in a solution of NaOH at different concentrations, 5%, 10% and 15% (*w*/*v*) for 24 h at a temperature of 23 ± 2 °C. The fibers were rinsed and dried again after the treatment.

#### 2.2.3. Nonwoven Formation

First, the alkali-treated banana fibers were blended with water to make a uniform pulp mixture. Then, they were rinsed thoroughly to remove any leftover NaOH and dried once again. The prepared banana pulp was placed in the blender at a fiber pulp/water ratio of 1:50. After blending, the mixture was poured into a mold prepared with a wooden frame and mesh fabric. The pulp fibers were distributed uniformly by immersing the complete mold on a tab of water according to the wet laid web formation technique. Then the distributed pulp web was moved to a plastic sheet and pressed with wiping paper to remove any excess water. Finally, the mixture was dried in sunlight and straightened with an electrical iron to remove any rough surfaces. A similar procedure was followed for all types of banana fiber nonwovens. The thickness of the nonwovens was found to be 0.75 ± 0.05 mm.

#### 2.2.4. Water Repellent Treatment of Nonwovens

Before making composites by reinforcing the nonwovens, some of them were treated with a water repellent (WR) chemical (perfluoroalkyl acrylate copolymer) to improve the hydrophobicity of the composites. WR was applied at three different concentrations, 5%, 10% and 15%, to determine the appropriate dose to decrease water absorbency while maintaining strength. The nonwovens were immersed in the WR solution and kept there for several minutes. The wet nonwovens were then squeezed by a padding roller to remove excess solution. They were then dried and cured in an oven at a temperature of 160–170 °C for 30 min.

#### 2.2.5. Fourier Transform Infrared (FTIR) Spectroscopy

FTIR spectrometer Perkin Elmer Spectrum was used at Institute of Materials Science, Kaunas University of Technology, Kaunas, Lithuania to observe the appearance of the chemical functional groups in raw banana fibers, alkali-treated banana fibers and water repellent-treated banana nonwovens by producing infrared absorption spectra. The FTIR spectra were investigated within a range of 4000–500 cm^−1^ (32 scans at 4 cm^−1^).

#### 2.2.6. Scanning Electron Microscopy (SEM)

The microstructure of the fiber surface was investigated by field emission scanning electron microscopy (FESEM) from FEI Quanta™ at Institute of Materials Science, Kaunas University of Technology, Kaunas, Lithuania. Samples were dried at 100 °C for 10 min to remove any inside moisture prior to testing.

#### 2.2.7. Composite Formation

The composites were prepared using the hand layup technique. The already-prepared nonwovens from four different sections of the banana tree, i.e., OB, MB, IB, and MR, were used as reinforcing material wile two types of resin, i.e., epoxy (E) and polyester (P), were used as a matrix. In total, eight variants of composite were prepared, which are designated as OB/E, MB/E, IB/E, MR/E, OB/P, MB/P, IB/P and MR/P, with all possible combinations of nonwovens and resins. Two metal plates were used as the top and bottom surfaces of the mold, with a size of 35 × 35 cm. The metal plates were wrapped with Teflon (PTFE) paper to avoid sticking difficulties during composite peel-off. Three layers of nonwovens were reinforced for all types of composites. At first, the nonwovens were cut to a size of 30 × 30 cm. Three pieces of nonwoven sheets were weighted together by a precise scale. According to the weight of the nonwovens, a certain amount of resin mixture was prepared with the addition of an appropriate catalyst by maintaining a constant fiber/resin weight ratio of 30:70 for all of the composites. For hardeners, 10% HY951 was used for epoxy resin and 2% MEKP was used for polyester resin. The bottom metal plate was placed on a suitable flat surface. To begin the fabrication of the composites, ¼ of the resin mixture was poured on the bottom metal plate and spread uniformly with a brush according to the size of the nonwovens. Then, the first nonwoven layer was placed on top and pressed with a hand roller in such a way that the resin penetrated throughout the nonwoven. Again, ¼ of the resin mixture was poured on the first nonwoven layer and the same process was repeated to reinforce the second and third nonwoven layers. The remaining ¼ of the resin mixture was poured on the third nonwoven layer, then the top metal plate was placed on them to make a complete sandwich structure. A dead weight of 20 kg was laid on the top metal plate and kept there 24 h for curing. Finally, the dead weight was removed and the composite was separated from the metal plates. A similar procedure was followed for making all of the composites. The overall thickness of the composites was found to be 3 ± 0.5 mm.

#### 2.2.8. Sampling

**Figure 2:**
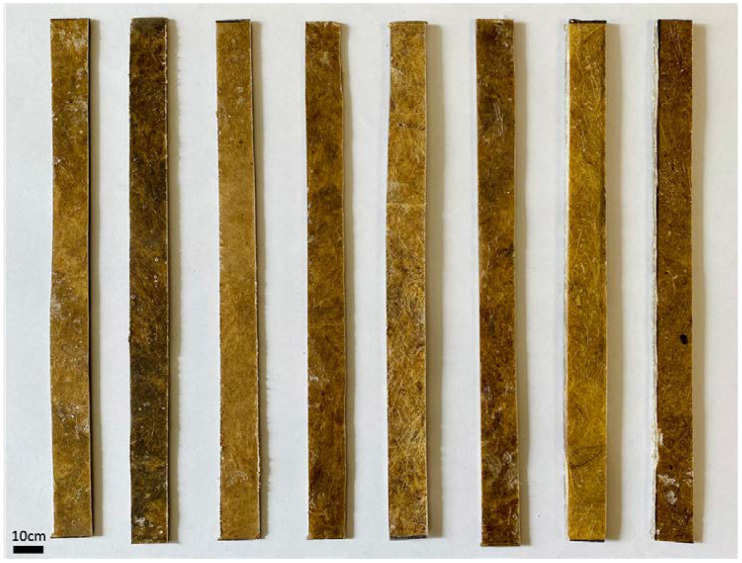
Prepared composite samples for the tensile test (from left: OB/P, OB/E, MB/P, MB/E, IB/P, IB/E, MR/P, MR/E).

Eight types of composites were prepared in total. For each test or treatment, samples were prepared separately according to the prescribed standards. Experiments were repeated five times for each type of test, and the number of samples was prepared accordingly. All types of sample are described in Table 1. Some prepared samples for the tensile tests are presented in Figure 2.

#### 2.2.9. Gamma Radiation on Composites

The composite samples were irradiated with different doses of gamma radiation to improve their mechanical properties. A capsule type of gamma irradiator, Co-60, was used at Institute of Radiation and Polymer Technology, Dhaka, Bangladesh. This irradiator has a remote-controlled electromechanical system with a capacity of 65Kci. Five different doses of gamma radiation were applied for each type of sample: 100 krd, 200 krd, 300 krd, 400 krd and 500 krd.

#### 2.2.10. Water Absorbency

Samples were prepared and analyzed according to the standard ASTM D570-98. Before immersion, they were conditioned in an oven for 24 h at 50 °C, cooled in a desiccator, and weighed immediately in order to determine the dry weight of each sample. The conditioned samples were then put in a beaker of water maintained at a temperature of 23 ± 2 °C. The samples were taken out for a maximum of 2 min to measure their weight at every hour for the first four hours, and then at every 4 h thereafter, for 24 h. Before measuring weight, the samples were wiped off every time to remove surface water. The water absorbency by weight percentage was calculated by the following Equation (1):

(1) $$Water\;Absorbency\;\left(\%\right)=\frac{W_{w}-W_{c}}{W_{c}}\times 100\;$$

where $W_{w}$ is wet weight after water immersion and $W_{c}$ is conditioned weight.

#### 2.2.11. Mechanical Tests

Tensile properties such as tensile strength (TS) and elongation at break percentage (Eb%) were tested according to the standard ASTM D638-14. A universal testing machine (UTM) from ZwickRoell was used for testing the samples at the Laboratory of Materials Engineering, Kaunas University of Technology (KTU), Kaunas, Lithuania. Samples were prepared according to the standard size of 165 mm × 13 mm. A gauge length of 50 mm was maintained. Load was applied at a constant rate of motion of 10 mm/min of grip. The tensile strength and elongation at break, respectively, were calculated by the following Equations:

(2) $$TS=\frac{F_{max}}{A}\;$$

where $F_{max}$ is maximum load and A is cross-sectional area of the sample;

(3) $$Eb\%=\frac{\Delta l_{b}}{l_{0}}\;$$

where $\Delta l_{b}$ is elongation at breaking point and $l_{0}$ is the initial length of the sample.

Flexural property was tested with the same UTM according to the standard ASTM D790-03 to determine the flexural strength (FS) of the composites. Samples were prepared according to the standard and placed on two supports with a span length of 16 times the thickness of the samples. Load was applied on the midspan with a constant deflection speed of 0.10 mm/mm/min until breaking. The flexural strength was calculated by Equation (4):

(4) $$FS=\frac{3FL}{2bd^{2}}\;$$

where *F* is breaking load in N, *L* is the length of the support span, *b* is width and *d* is the thickness of the sample.

## 3. Results and Discussions

### 3.1. Fiber Characterization

#### 3.1.1. FTIR Spectroscopy Analysis

**Figure 3:**
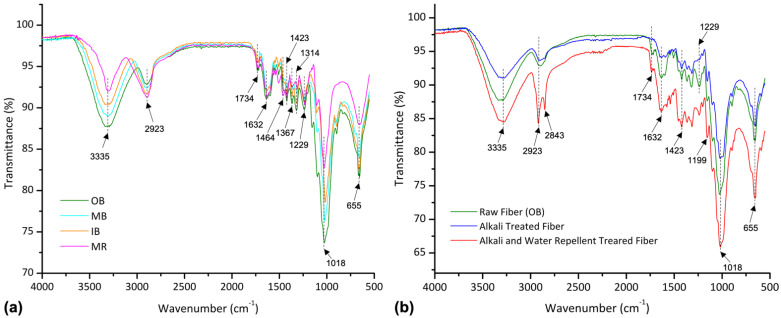
FTIR spectroscopy of (**a**) different types of raw banana fibers, (**b**) raw, alkali-treated, and water repellent-treated banana fiber nonwovens.

Figure 3 presents the FTIR spectroscopy of different types of raw banana fibers as well as the changes after alkali and water repellent treatments. On the left, IR spectroscopy of raw banana fibers (i.e., OB, MB, IB and MR) shows quite similar major peaks except for some differences in intensity in the range 3400 cm^−1^ to 600 cm^−1^. To begin with, the peak at 3335 cm^−1^ corresponding to O-H stretching in the carboxylic acid group of cellulose was found for all four types of fibers. The higher intensity for OB and lower for MR at this peak defines the highest and lowest potential amount of cellulose content, respectively [40]. Moreover, hydrogen and covalent bonds are developed with these hydroxyl groups by intra- and intermolecular crosslinks, with water bonding at wavenumber 3400–3000 cm^−1^ [41]. The peak at 2923 cm^−1^ corresponds to CH2 asymmetric stretching, perhaps due to the appearance of hemicellulose [40]. The intensity at this peak shows the possibility of lower hemicellulose in OB and higher hemicellulose in MR banana fibers. Another major peak at 1632 cm^−1^ is due to the aromatic C=C in plane alkenes, showing the presence of lignin. Peaks observed at wavenumber 1464 cm^−1^ due to -C-H stretching also reveal the appearance of lignin. From the intensity, it can be said that OB and MR fiber contain more lignin than the others. Wavenumbers 1420–1430 cm^−1^ are related to the amount of crystallinity in cellulose [41]. Among the four types of fibers, OB contains a more oriented structure of cellulose, as shown by analyzing the fibers’ respective intensity. The highest intensity for all the fibers at 1018 cm^−1^ represents the presence of ester C-O stretching.

Figure 3b shows the IR spectroscopy of alkali-treated and water repellent-treated OB fiber. The higher intensity of the raw fiber compared to alkali-treated fiber indicates the presence of different unwanted materials such as natural minerals, oil, and wax in the cellulosic compounds, which were removed by alkali treatment. IR spectroscopy also reveals that the amount of other chemical constituents like hemicellulose (at 2923 cm^−1^), lignin (at 1632 cm^−1^) and other alkali soluble compounds were decreased by strong NaOH treatment. Peaks at 1734 cm^−1^ responsible for C=O (aldehyde) and 1229 cm^−1^ responsible for C-OH which were observed in raw fiber do not appear after alkali treatment.

However, IR spectroscopy of OB fiber after water repellent treatment shows some additional peaks with high intensity. For example, at wavenumber 2843 cm^−1^ methoxy (O-CH_3_) groups and C-H stretching can be observed, indicating the presence of inorganic compounds, in this case water repellent. In addition, the high intensity peaks at 655 cm^−1^ (responsible for the deformation vibration of -CF_3_) and at 1199 cm^−1^ belong to symmetric -CF_2_ (Perfluoro methylene), which indicates the appearance of fluorine-based WR in the fiber. Moreover, the reappearance of aldehyde groups at 1734 cm^−1^ (which were removed after alkali treatment) reveals the presence of the surface treatment. Overall, the higher intensities after WR treatment indicate the addition of functional groups and possible coating on the fiber surface, which can improve the hydrophobicity of the fibers.

#### 3.1.2. Scanning Electron Microscopy (SEM) Analysis

**Figure 4:**
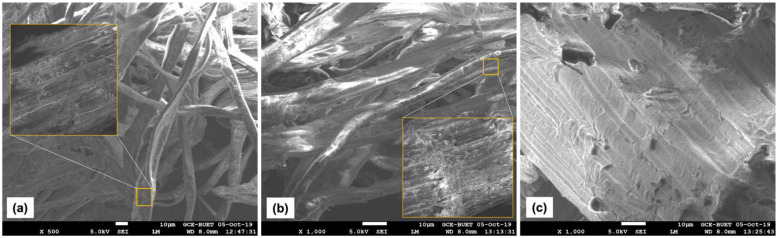
SEM micrograph of (**a**) raw banana fibers, (**b**) alkali treated banana fibers, and (**c**) water repellent-treated banana fiber nonwovens.

Samples were analyzed by SEM in three stages as raw fiber, after alkali treatment, and after water repellent treatment. Micrographs from SEM are presented in Figure 4. The first micrograph shows the microscopic surface view of raw banana fibers. Beside their main constituents, the banana fibers also contain some organic matter and natural impurities like oil, wax, and minerals that usually fill their porous cellulosic structure. Because of this, the fiber surface can be found quite smooth, as shown in Figure 4a. Nevertheless, there can be also found slightly rougher surface, as shown in Figure 4a. The micrograph also reveals the irregularities in the morphological orientation of the fiber bundles, which indicate the presence of amorphous regions on the fibers.

The microscopic view of the alkali-treated fiber surface is presented in Figure 4b. Because of the strong NaOH treatment, impurities such as oil and wax as well as some amounts of hemicellulose, lignin and other soluble compounds are removed, which creates the rougher fiber surface seen in this micrograph. The effect of NaOH is clearer in the magnified window of Figure 4b. However, this rough fiber surface confers the advantage of strong interlocking among the fibers. Especially for the manufacturing of nonwovens, this strong mechanical anchoring makes them stronger, and for the manufacturing of fiber-reinforced composites it increases fiber–matrix adhesion.

A water repellent chemical was also applied to the surface of nonwovens made of banana fibers. From the micrograph presented in Figure 4c, WR created a thin layer on the fiber surface instead of filling the pores, due to its low molecular weight. As the WR was applied on the nonwoven surface, this thin coating did not interrupt fiber interlocking while making the nonwoven; however, it may still interrupt fiber–matrix adhesion while making the composites.

### 3.2. Water Absorbency

#### 3.2.1. Effect of Alkali Treatment on Water Absorbency

**Figure 5:**
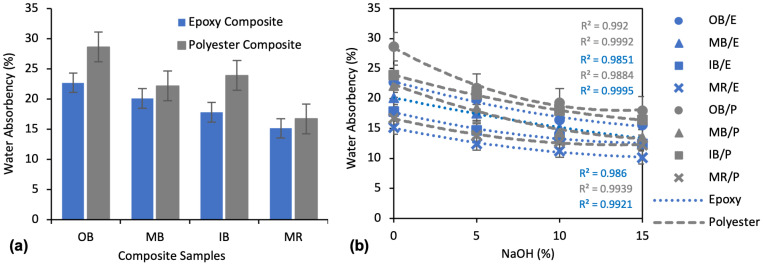
Water absorbency percentage of composites after 24 h water immersion: (**a**) Water absorbency of untreated (0% NaOH) composite samples; (**b**) Effect of alkali treatment on the water absorbency of the composite samples.

Figure 5a demonstrates the water absorbency of different types of banana nonwoven composites after 24 h of water immersion. The effects of alkali treatment in three different concentrations, 5%, 10% and 15% (*v*/*w*) of NaOH, on the water absorbency of the composites are presented in Figure 5b. Among the untreated composites, MR/E shows the lowest water absorbency, at 15.14%, and OB/P shows the highest absorbency, at 28.63%. OB and MB composites exhibit higher absorbency and MR composites exhibit lower absorbency for both polyester and epoxy matrix. In comparing epoxy and polyester composites, epoxy always showed lower absorbency than polyester composites. For instance, OB/E, MB/E, IB/E and MR/E were found to have 20.7%, 9.28%, 25.43% and 9.45% lower water absorbency than OB/P, MB/P, IB/P and MR/P, respectively.

The water absorbency of the composites varied with different types of banana fibers. This may be due to the different chemical composition percentage of the banana fibers, as they are collected from different parts of the banana tree. The chemical analysis (FTIR) of the fibers also proves the variation in chemical composition. Moreover, from the earlier studies, the chemical compositions of the banana stem fibers also varied noticeably, with different percentages of cellulose content: 71.08% [13], 60–65% [42], 57.6% [43], and 43.46% [29]. The other contents, such as lignin and hemicellulose, also varied remarkably in these studies. As discussed above, the OB nonwoven showed highest intensity of hydrophilic O-H stretching and was easily crosslinked with the hydrogen ions of water. Thus, the more common hydrophilic sites of the OB composite are responsible for its higher absorbency. The composites made with epoxy matrix showed less absorbency than those made with polyester matrix. This may be due to the better interfacial adhesion between fiber and epoxy matrix than polyester [22], which results in better coverage of hydrophilic fibers by the hydrophobic resins and makes them more watertight. Better adhesion also leads to removal of the amorphous regions and greater porosity in the fiber–matrix interface, and thus, water absorbency is reduced.

Alkali treatment improved the hydrophobicity of the composite samples, as can be clearly seen in Figure 5b. The water absorbency of all the composites was decreased significantly for all the fibers to which the NaOH concentration was applied. For example, at the lowest NaOH treatment concentration of 5%, the OB/E, MB/E, IB/E, and MR/E composites saw decreased absorbency of 12.5%, 12.9%, 17.7% and 17.9%, respectively. Decreases of 24.1%, 17.1%, 13.6% and 17.2% were found for the OB/P, MB/P, IB/P and MR/P composites, respectively, with the same 5% NaOH treatment. An even greater influence was found with the 10% NaOH treatment. For instance, a 27.5%, 24.8%, 23.7%, 25.6% decrease in water absorbency was found for the OB/E, MB/E, IB/E, MR/E composites, respectively, with a respective decrease of 32.7%, 35.2%, 25.0%, 23.7% for the OB/P, MB/P, IB/P, MR/P composites, compared to untreated composites.

Water absorbency continued to decrease at 15% NaOH as well. From the second-order polynomial curve, it is evident that the influence of NaOH is lower with the 15% than with the 10% concentration. Between the epoxy and polyester composites, the latter were influenced more by the NaOH than the former. For example, with the 10% NaOH treatment, water absorbency was decreased by an average of approximately 30% for Polyester composites and 22% for Epoxy composites.

The hydrophobicity of the composites was improved with the various concentration of alkali treatments. This is because alkali treatment removes unwanted materials such as pectin, oil, wax, lignin, hemicellulose and other impurities to a certain degree [25]. This results in better adhesion between the fiber and matrix. This stronger interfacial bonding means that the fibers are firmly protected within the hydrophobic matrix, thus decreasing their water absorbency [26]. The application of NaOH also reduces the hydroxyl groups of cellulose (the main group responsible for absorbing water) by ionizing them into alkoxides [44,45].

**Figure 6:**
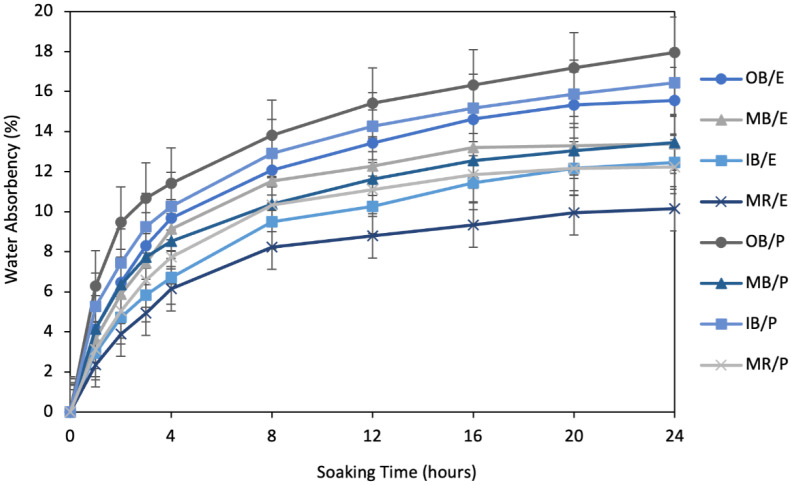
Water absorbency flow of 15% NaOH-treated composite samples, by soaking time up to 24 h.

Water absorbency flows, as described in Figure 6, show that the water absorbency rate is very high in the first several of hours. It can be observed that approximately 40–50% of the water was absorbed in the first two hours of the 24 h of complete observation. The rate can be considered medium during time 3–8 h, with about 80% of the water absorbed after 8 h of 24 h. Subsequently, all of the flows became slow up to 20 h, and very slow after 24 h.

#### 3.2.2. Effect of Water Repellent Treatment on Water Absorbency

**Figure 7:**
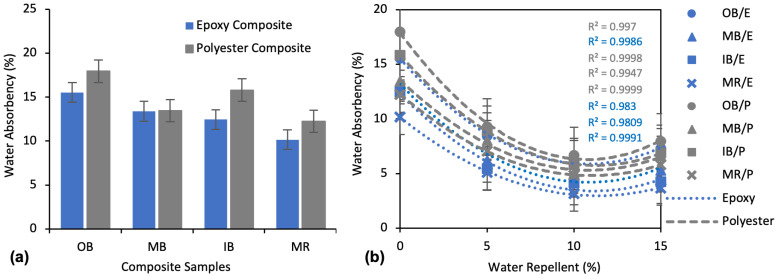
Water absorbency percentage of composites after 24 h water immersion: (**a**) Water absorbency of untreated (0% WR, 15% NaOH) composites; (**b**) Effect of WR treatment on the water absorbency of the composites.

The water absorbency of untreated WR (0% WR + 15% NaOH) composites after 24 h of immersion in water is presented in Figure 7a. The OB/P composite showed the highest water absorbency at 15.55%, while the MR/E composite showed the lowest at 10.16%. OB composites exhibited higher absorbency than all the other types of composites for both epoxy and polyester composites. Between the two types of matrixes, epoxy composites showed significantly lower water absorbency than polyester composites, except for MB composites where the absorbency was similar. For instance, OB/E, MB/E, IB/E and MR/E were found to have 13.44%, 0.43%, 21.16% and 17.03% lower water absorbency than OB/P, MB/P, IB/P and MR/P, respectively.

For the further improvement of hydrophobicity, alkali-treated nonwovens were treated again with water repellent chemicals. Figure 7b details the effects of WR on the water absorbency of the composites. It is evident that WR treatment decreased the water absorbency remarkably for all types of composites. At a concentration of only 5%, WR treatment reduced the water absorbency by 45.7%, 53.8%, 56.3%, 49.9% for OB/E, MB/E, IB/E, and MR/E composites and 48.3%, 40.4%, 43.5%, 42.4% for OB/P, MB/P, IB/P, and MR/P composites, respectively, as compared to untreated composites. Water absorbency continued to decrease further with application of 10% WR. An overall 60–70% decrease of water absorbency was found at 10% WR compared to untreated; however, from the second order polynomial curves the influence is lesser by 10–20% when compared to 5% WR.

One the other hand, water absorbency started to increase at 15% WR for all the composites. The absorbency increased by 7.7%, 3.5%, 0.9%, 5.0% for OB/E, MB/E, IB/E, and MR/E and by 7.1%, 11.6%, 6.9%, 8.0% for OB/P, MB/P, IB/P, and MR/P composites, respectively, compared to absorbency at 10% WR.

WR treatment improves the hydrophobicity of the composites dramatically, to a certain level of concentration. The WR chemical used in this study was perfluoroalkyl acrylic. This WR creates a surface coating on the materials and consequently prevents water molecules from entering the inside of the material. WR may also crosslink with cellulose to make a harder and rougher surface. This rougher surface of the fiber creates air traps on the surface that increase hydrophobicity [46,47]. However, at 15% WR the water absorbency started to increase, which is due to the thicker coating on the fiber surface. This weakens the interfacial fiber–matrix bonding and allows water to penetrate inside the structure.

**Figure 8:**
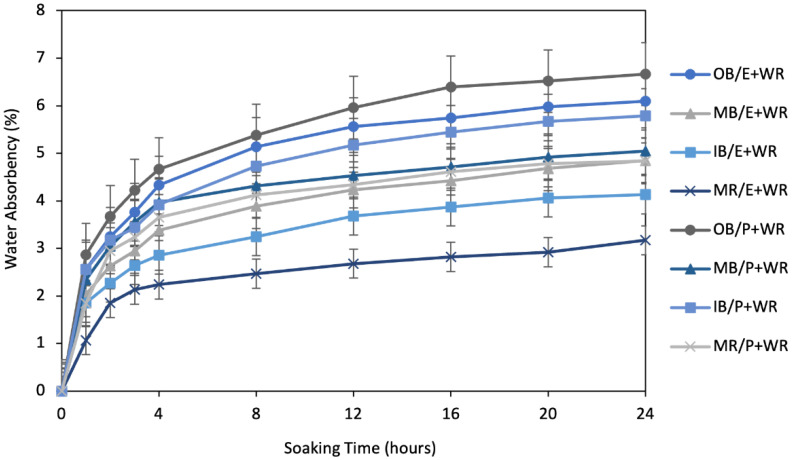
Water absorbency flow of 10% WR treated composite samples, by soaking time up to 24 h.

From the absorbency flow over the soaking times presented in Figure 8, water absorbency was very fast in first couple of hours, similar to the absorbency flows of the alkali treated composites. About 50–60% of water was absorbed in first two hours and about 75–85% in the first eight hours. After this time, all the flows became very slow. Moreover, at 24 h, they seemed quite stable, having absorbed the maximum amount of water. The main difference between the flows after alkali treatment (Figure 6) and after WR treatment (Figure 8) is that the curves look more stable at 24 h after WR treatment than after alkali treatment. This provides an assumption of further water absorbency of the composites after the period of 24 h. The composites after alkali treatment will continue to take in water over a long period of time, whereas the composites after WR treatment will stop taking water shortly after the period of 24 h has elapsed.

### 3.3. Mechanical Properties

Mechanical properties such as Tensile Strength (TS), Flexural Strength (FS) and Elongation at Break (Eb%) and the effects of alkali treatment, water repellent treatment and gamma radiation on the mechanical properties were investigated for all of the composites.

#### 3.3.1. Effects of Alkali Treatment

##### Effect of Alkali Treatment on Tensile Strength

**Figure 9:**
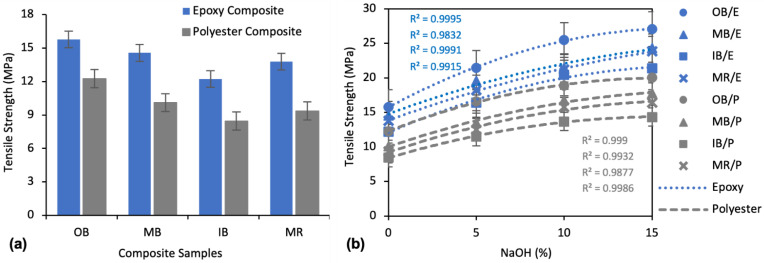
(**a**) Tensile strength of untreated (0% NaOH) composites; (**b**) Effect of alkali treatment on the tensile strength of the composites.

The tensile strengths of the untreated (0% NaOH) composites are illustrated in Figure 9a. It can be seen that OB/E exhibits the highest TS (15.78 MPa) and IB/P exhibits the lowest TS (8.45 MPa) among all types of composites. The different fiber types which were used for making the nonwovens and subsequent composites had an influence on tensile strength. Among them, tensile strength was found to follow a sequence of OB > MB > MR > IB for both epoxy and polyester matrix composites, with 15.78 MPa, 14.56 MPa, 13.78 MPa, and 12.23 MPa being found for OB/E, MB/E, MR/E, and IB/E and 12.25 MPa, 10.11 MPa, 9.35 MPa, and 8.45 MPa was found for OB/P, MB/P, IB/P, and MR/P composites, respectively. Among the two types of matrices, epoxy composites demonstrated higher tensile strength than polyester composites. OB/E, MB/E, IB/E and MR/E composites were found to have a 28.8%, 44.1%, 44.7% and 47.4% higher TS than OB/P, MB/P, IB/P and MR/P composites, respectively.

There is an apparent influence of alkali treatment on the tensile strength of the composites, as shown in Figure 9b. It is evident that TS increased with the increase of NaOH concentration. With 5% NaOH treatment, TS was increased by about 35%, whereas an increase of about 60% was found at 10% NaOH and a 75% increase at 15% NaOH treatment, on average, for all types of composites. For instance, at the maximum 15% NaOH treatment, TS was increased by 71.4%, 67.1%, 74.8%, and 72.9% for OB/E, MB/E, IB/E, and MR/E composites and by 63.3%, 75.7%, 69.5%, and 75.8% for OB/P, MB/P, IB/P, and MR/P composites, respectively. The second order polynomial curves prove that the impact of alkali treatment was greater with the 5% and 10% concentrations than with 15% concentration.

##### Effect of Alkali Treatment on Flexural Strength

**Figure 10:**
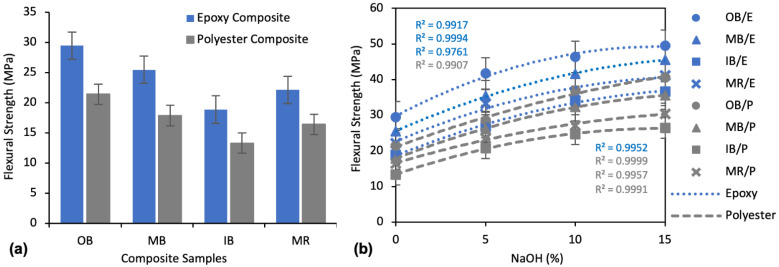
(**a**) Flexural strength of untreated (0% NaOH) composites; (**b**) Effect of alkali treatment on flexural strength of the composites.

Figure 10a shows the flexural strengths (FS) of the untreated composites, and the effect of alkali treatment on flexural strength is shown in Figure 10b. Flexural strength was found to follow a sequence of OB > MB > MR > IB for both epoxy and polyester matrix composites. The highest FS, 29.47 MPa, was found for OB/E composite and the lowest, 13.32 MPa, for the IB/P composite. The OB/E composites exhibited a 56.1% higher FS than IB/E, and the OB/P composites exhibited a 60.1% higher FS than IB/P, which clearly defines the effects of fiber types from different parts of banana trees on the FS of the composites. As with TS, epoxy composites showed better FS than polyester composites. OB/E, MB/E, IB/E and MR/E composites were found to have a 37.5%, 42.4%, 41.7% and 34.7% higher TS than OB/P, MB/P, IB/P and MR/P composites, respectively.

The effect of alkali treatment on the flexural properties of the composites followed a similar trend as the tensile properties described earlier. Flexural strength increased with increased NaOH concentration. For example, at a concentration of 10% NaOH FS was improved by 47.3%, 63.3%, 80.8%, and 64.1% for OB/E, MB/E, IB/E, and MR/E composites and by 72.4%, 80.8%, 85.2%, and 65.2% for OB/P, MB/P, IB/P, and MR/P composites, respectively, compared to untreated composites. The improvement was even more apparent at a concentration of 15% NaOH. From the second order polynomial curve, the impact was greater at 10% NaOH than at 15%, considering the amount of NaOH.

##### Effect of Alkali Treatment on Elongation at Break

**Figure 11:**
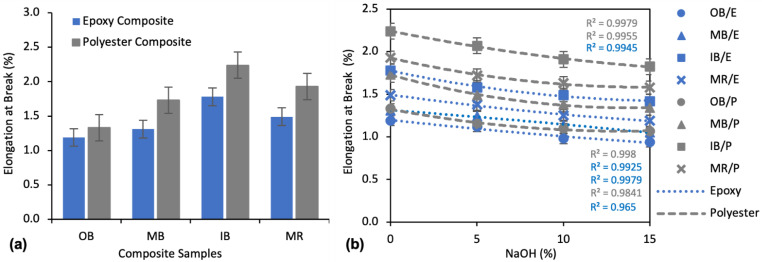
(**a**) Eb% of untreated (0% NaOH) composites; (**b**) Effect of alkali treatment on Eb% of the composites.

The elongation properties of the composites showed an exactly opposite trend to TS and FS, as presented in Figure 11a. The highest Eb% was found for IB/P and lowest for OB/E. In comparing the different nonwoven reinforcements, OB always showed the lowest and IB always showed the highest Eb%, while MB and MR exhibited medium Eb% for both polyester and epoxy matrix composites. The composites with a polyester matrix showed higher Eb% than those with an epoxy matrix. OB/P, MB/P, IB/P and MR/P demonstrated an 11.8%, 32.1%, 25.8% and 29.5% higher Eb% than OB/E, MB/E, IB/E and MR/E composites, respectively.

The effects of NaOH on the Eb% of the composites are illustrated in Figure 11b. Alkali treatment reduced the Eb% to a small extent. A maximum reduction of 21.0%, 19.8%, 20.2%, and 20.1% Eb% was found for OB/E, MB/E, IB/E, and MR/E composites, and 19.9%, 22.5%, 18.5%, and 18.1% for OB/P, MB/P, IB/P, and MR/P composites, respectively, at a concentration of 15% NaOH.

After analyzing the mechanical properties of the composites for TS, FS and Eb%, the results can be summarized thusly: OB composites showed better TS and FS but lower Eb% than the other types of nonwoven composites, while IB composites exhibited lower TS and FS but higher Eb%. MR and MB can be considered medium in all cases. As discussed above, there can be some variation in the chemical compositions of different types of banana fibers. From the FTIR analysis, OB nonwovens contain a higher percentage of cellulose and a greater possibility of inter- and intramolecular hydrogen bonding. This composition is related to large polymeric chains of cellulose with crosslinking among them, and makes for very good adhesion with the matrices by crosslinking with the polymer of the matrix, which makes the resulting composites stronger. Moreover, FTIR shows the lignin content is also higher in OB nonwovens. Though some lignins are removed by alkali treatment, the rest of them can link with hemicellulose by covalent bonding, strengthening the structure of cellulose–hemicellulose [48]. Furthermore, OB nonwovens appear more crystalline from the perspective of some specific peaks in FTIR, which gives them a more solid and well-oriented structure than other types of fibers. On the other hand, the cellulose percentage as well as the capability for intra- and intermolecular crosslinking may gradually decrease from the outer bark (OB) to the inner bark (IB) of the banana tree. Thus, the smaller polymeric chains and lower adhesion with the matrix gradually decrease the mechanical properties of MB and IB. From the chemical analysis, MR nonwovens showed higher amounts of lignin and hemicellulose rather than cellulose, which can make them slightly stronger than IB. Previous studies have also proven the superior mechanical properties of OB as a nonwoven material [49]. Epoxy composite always resulted in better mechanical properties, e.g., higher values of TS and FS and lower Eb% than polyester composites. This is because of better interfacial bonding between fiber and epoxy, which leads to very good adhesion between them. As a result, the applied load can be distributed properly through the fiber and matrix, which leads to greater loadbearing ability. Similar results have been found in some previous studies [22,50,51].

Alkali treatment resulted in the improvement of TS and FS and decrease in Eb%. As discussed above, alkali treatment eliminates some unwanted materials, including lignin and hemicellulose. This elimination creates rough fiber surfaces that lead to better mechanical interlocking among the fibers. By cleaning the impurities the cellulose content of the fibers is increased, which may increase the reactive sites and create strong bonding with the matrix. Therefore, the mechanical properties like TS and FS were improved. For the same reason, the Eb% of the composites was decreased. With better adhesion between the fiber and matrix, the material become more solid and hard, and thus the elongation property declines [26,27,28,44].

#### 3.3.2. Effects of Water Repellent

##### Effect of Water Repellent Treatment on Tensile Strength

**Figure 12:**
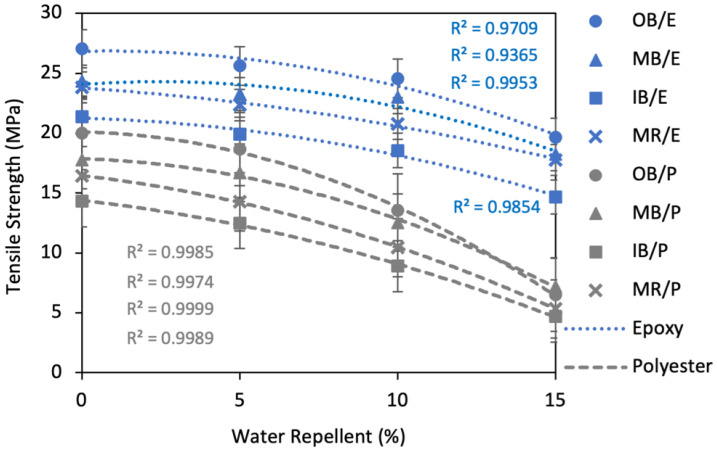
Effect of water repellent on the tensile strength of the composites.

Figure 12 reveals the effects of WR on the tensile strength of the composites. There is no doubt that the hydrophobicity of the composites was improved to a great extent by the WR treatment. On the other hand, this treatment had a negative effect on the tensile properties of the composites. A maximum decrease in TS of 27.3%, 25.1%, 31.4% and 25.4% was found for OB/E, MB/E, IB/E and MR/E composites, respectively, compared to a maximum decrease of 67.5%, 59.4%, 67.3% and 67.5% for OB/P, MB/P, IB/P and MR/P composites, respectively. However, this deterioration was very low (about 4–14%) at 5% WR concentration. For instance, the TS of the OB/E, MB/E, IB/E and MR/E composites was decreased by 5.3%, 4.4%, 7.0% and 6.2%, whereas the TS of OB/P, MB/P, IB/P and MR/P composites was decreased by 6.7%, 6.0%, 12.9% and 13.2%, respectively, at a concentration of 5% WR. At 10% WR, the TS of the epoxy composites was reduced by approximately 10% on average, whereas the TS of the polyester composites was reduced by approximately 34% on average. Likewise, at 15% WR, polyester composites also showed higher reduction (approximately 65%) than epoxy composites (approximately 27%) on average.

##### Effects of Water Repellent Treatment on Flexural Strength

**Figure 13:**
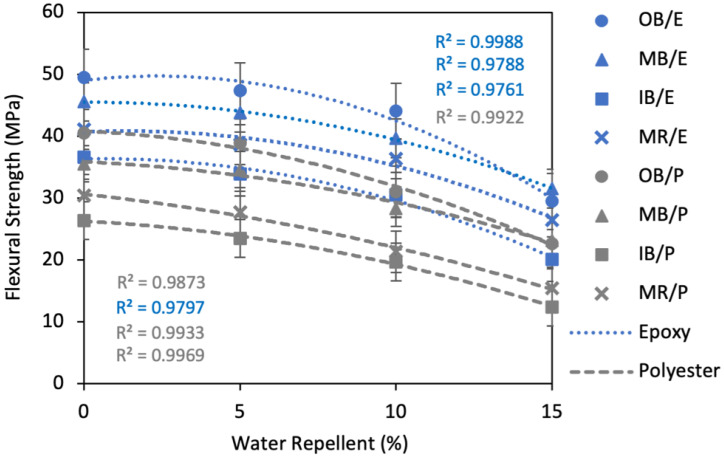
Effect of water repellent on the flexural strength of the composites.

The effect of WR on the flexural properties of the composites are evident from Figure 13. A similar negative trend was found for all the composites; like TS, it declined to a large extent (about 40–50% on average) after treatment with 15% WR, as compared to the untreated composites. However, the effect was much lower at 5% and 10% WR. For example, the FS of OB/E, MB/E, IB/E and MR/E composites declined by 4.4%, 3.9%, 7.5% and 6.0%, while the FS of OB/P, MB/P, IB/P and MR/P composites declined by 4.1%, 2.6%, 11.0% and 8.8%, respectively, at 5% WR when compared to untreated composites.

##### Effects of Water Repellent Treatment on Elongation at Break

**Figure 14:**
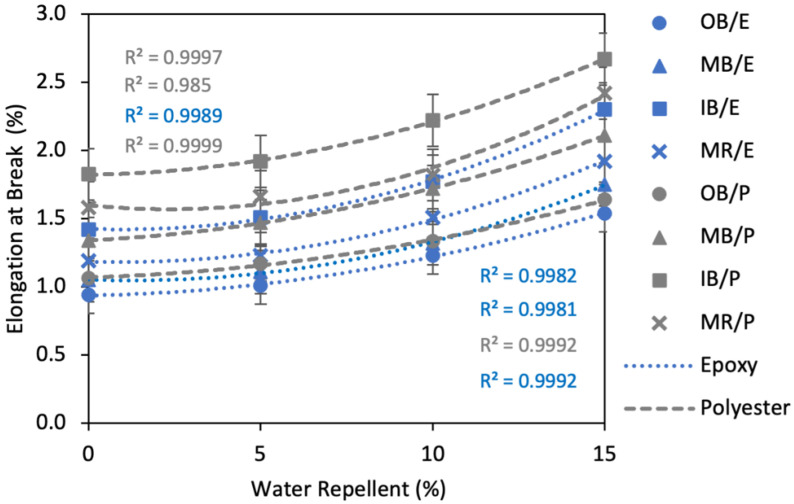
Effect of water repellent on elongation at break (%) of the composites.

Elongation of the composites increased with increased WR%, as clearly shown in Figure 14. The maximum increase of Eb% was found at 15% WR. For example, OB/E, MB/E, IB/E and MR/E composites exhibited a 63.8%, 66.7%, 62.0% and 61.3% increase, respectively, at 15% WR compared to untreated composites. At 10% WR, the effect was lower; Eb% increased by about 25% on an average for all types of composites. Furthermore, at 5% WR the effect was very low, as Eb% was increased by a maximum of about 10% compared to the untreated composites. The second order polynomial curves also prove this trend.

The application of WR on the nonwoven surface decreased the mechanical properties of TS and FS and increased Eb%. This was expected as WR creates a coating on the fiber surface which resist water and prevents it from penetrating inside the fiber, thus improving hydrophobicity. However, because of this coating or polymer blockage, mechanical properties can be reduced as the fiber–matrix interface is disrupted and the adhesion between the fiber and matrix is weakened, resulting poorer mechanical properties. Nevertheless, this effect is negligible at lower concentrations such as 5% WR. Our study reveals that the deterioration of TS and FS is by a maximum of about 10% for all types of composites at a concentration of 5% WR. On the other hand, the increase in the hydrophobicity is about 40–50% with the same concentration of WR. Therefore, application of WR at a concentration of 5% is recommended in order to balance water absorbency and mechanical properties.

#### 3.3.3. Effects of Gamma Radiation

The effects of gamma radiation on mechanical properties like tensile strength, flexural strength and elongation at break were investigated in this study. The results are described in second order polynomial curves because gamma radiation influenced the mechanical properties by two opposite factors.

##### Effect of Gamma Radiation Treatment on Tensile Strength

**Figure 15:**
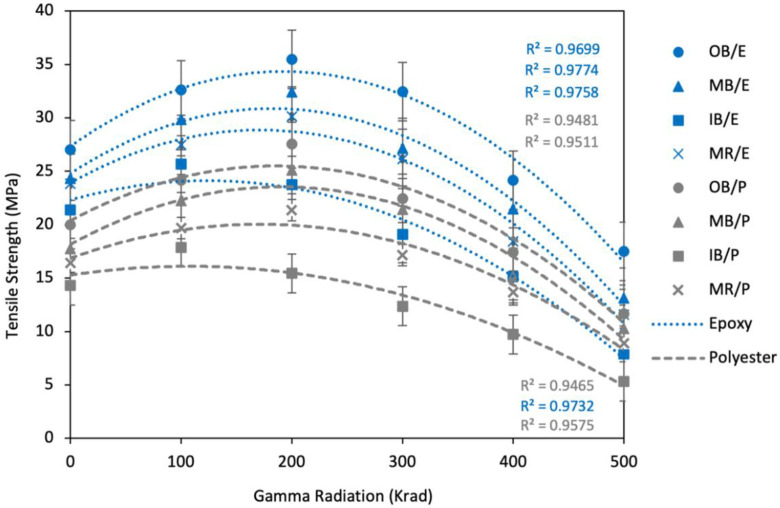
Effect of gamma radiation on tensile strength of the composites.

Figure 15 depicts the influence of gamma radiation on the tensile properties of the composites. All the curves demonstrate that gamma radiation improves the mechanical properties significantly to a certain level of dose. The TS of the OB/E, MB/E and MR/E composites were improved by a maximum of 31.2%, 33.3% and 37.7% at a gamma radiation dose of 200 krd, whereas the TS of IB/E was improved by 20.1% at a dose of 100 krd, respectively, compared to non-irradiated composites. However, the TS of the composites was decreased to a large extent by further increasing the gamma radiation dose. For instance, the TS of OB/E, MB/E, and MR/E composites was decreased by 8.5%, 16.3%, and 13.1% at 300 krd, and the TS of IB/E was decreased by 7.4% at 200 krd, respectively, compared to the maximum value. A similar trend was found for polyester composites, with an increased in TS by 37.7%, 41.4%, and 30.0% for OB/P, MB/P, and MR/P composites at 200 krd, and 24.9% for IB/P composites at 100 krd, respectively; however, after this the TS decreased with increased radiation dose. At higher doses such as 500 krd, TS decreased drastically, by 60% on average for all types of composites from the maximum value of TS, which is less than half the TS of even nonirradiated composites. Both the polyester and epoxy composites were influenced by gamma radiation in the same way, though the TS of epoxy composites was improved to a slightly higher degree than polyester composites.

##### Effect of Gamma Radiation Treatment on Flexural Strength

**Figure 16:**
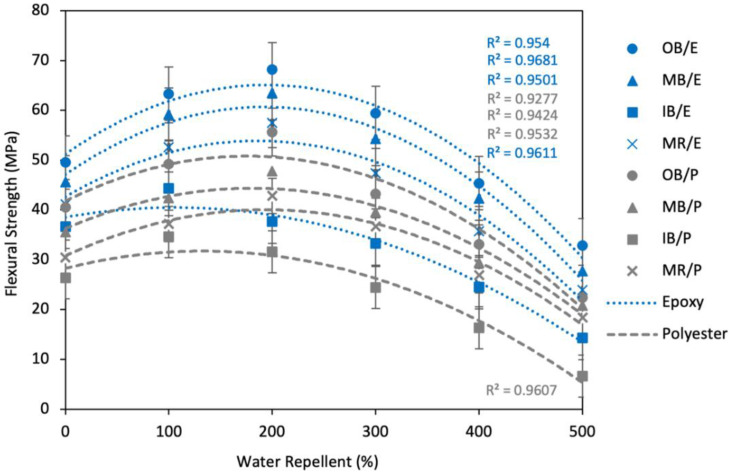
Effect of gamma radiation on the flexural strength of the composites.

The effect of gamma radiation on the flexural strength of the composites is presented in Figure 16. A similar tendency to TS was found in this case as well; the FS of the composites was enhanced noticeably up to a certain dose, then reduced by a large amount above that level. It is evident that FS was improved by 37.8%, 39.2%, and 39.8% for OB/E, MB/E, and MR/E composites and by 37.3%, 34.4%, and 40.8% for OB/P, MB/P, and MR/P, respectively, at a gamma radiation dose of 200 krd. IB composites such as IB/E and IB/P showed the maximum 21.1% and 31.2% improvement in FS at a radiation dose of 100 krd. Further increases in gamma dose, for example at 300 krd, saw FS decreased by 12.9%, 14.5%, 25.0%, and 17.6% for OB/E, MB/E, IB/E, and MR/E composites and 22.3%, 17.2%, 29.4%, and 14.3% for OB/P, MB/P, IB/P, and MR/P composites, respectively. FS fell dramatically at higher doses such as 500 krd. FS was found to be 32.89, 27.69, 14.34 and 23.92 MPa for OB/E, MB/E, IB/E and MR/E composites, respectively, and 22.38, 20.79, 6.67 and 18.36 MPa for OB/P, MB/P, IB/P and MR/P composites, respectively, at a gamma dose of 500 krd, which is almost half the FS of nonirradiated composites.

The improvement of TS and FS through gamma radiation is mainly due to improved polymeric bonding in the intra-chain of fiber and matrix by cross-linking to each other. This leads to more well-oriented polymeric structure and higher fiber–matrix adhesion, and therefore increases mechanical properties like TS and FS [32,52]. Gamma radiation is a powerful type of ionizing radiation which has the ability to penetrate materials and influence their polymeric structure by producing reactive species such as free radicals, ions, and peroxides. Consequently, these reactive species can cross-link or bind to each other to form long polymeric chains or large molecules, leading to changes in the mechanical properties of the materials. It is also evident from several studies that gamma radiation can break the C=C bond and generate free radicals, subsequently improving mechanical properties [32,34,35]. Gamma radiation may also extract the inside moisture of the composites, which is a possible reason for improvement of their properties [33,35].

In spite of this, mechanical properties like TS and FS began to decrease after a certain level of dose. This reduction is because of another aspect usually known as chain scission or chain degradation, which is the complete opposite of chain cross-linking. At higher doses, the main polymeric chains are broken down into small particles and the mechanical properties like TS and FS are thus decreased [32].

##### Effect of Gamma Radiation Treatment on Elongation at Break

**Figure 17:**
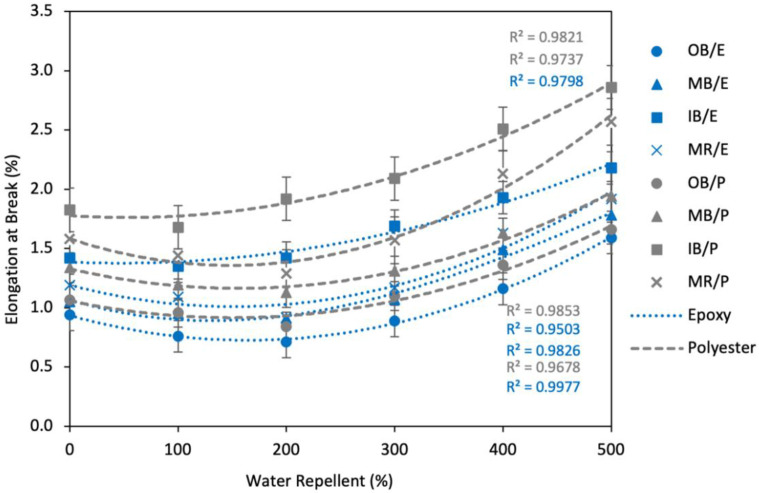
Effect of gamma radiation on elongation at break (%) of the composites.

The effect of gamma radiation on the Eb% of the composites is revealed in Figure 17. The Eb% was reduced by the gamma radiation to a small amount up to a certain level of irradiation, then increased gradually. At a dose of 200 krd, Eb% was decreased by a maximum of 20% from the nonirradiated composites, considering all types of composites, except for IB composites which exhibited the lowest Eb% at 100 krd. After this point, EB% increased with the increment of gamma radiation doses. The highest Eb% was found at a radiation dose of 500 krd, with 1.59, 1.78, 2.18 and 1.92% for OB/E, MB/E, IB/E and MR/E composites, respectively, and 1.66, 1.94, 2.86 and 2.57% for OB/P, MB/P, IB/P and MR/P composites, respectively; these values are 80–90% higher than nonirradiated composites.

As described above, gamma radiation leads to strong cross-linking among the intra-chains of fibers and matrices and ensures better adhesion among them. The resulting polymeric structures become more crystalline and limit the movement of polymer chains, which leads to a lower Eb% of the materials [53]. In other words, the more well-oriented structure makes the materials more solid and hard, and reduces the elongation properties. However, at higher doses the main polymer chains and fiber–matrix bonding may break down into small pieces resulting in severe disordering of the polymeric structure and thus higher elongation.

## 4. Conclusions

The current study reveals the development of innovative natural composite materials by reinforcing different banana fiber nonwovens, developed by a special manual technique of wet laid web formation. The outcome of this study can be summarized by the following points:

- OB composites showed higher mechanical properties (TS and FS) and higher water absorbency than other nonwoven composites due to the variation in chemical composition, which was pointed out by FTIR analysis. Between the two matrices, polyester composites exhibited higher absorbency and lower mechanical properties than epoxy composites.
- The hydrophobicity and mechanical properties of the composites were improved significantly by alkali treatment, due to the better fiber-matrix adhesion which is achieved through this treatment by removing unwanted materials from the fibers. For instance, an average decrease of about 32% in water absorbency and average increase of 71% in TS and 87% in FS was found at a concentration of 15% NaOH.
- Hydrophobicity continued to improve remarkably with water repellent treatment on the nonwovens by creating a surface coating on the materials. On the other hand, the mechanical properties were decreased as a result of disrupting the fiber matrix bonding through this treatment. This decrease was generally less than 10% on average at a concentration of 5% WR, with a significant improvement of hydrophobicity, by 47.5% on an average. Therefore, we recommend applying WR at a maximum concentration of 5% in order to balance water absorbency and mechanical properties.
- Gamma Radiation improved mechanical properties like TS and FS and decreased Eb%, thanks to the more well-oriented polymeric structure achieved using radiation. A maximum increase of 30% in TS and 35% in FS was observed at a radiation dose of 200 krd; however, further increasing the dose decreased these properties due to breaking of the main polymeric chains by higher radiation. Thus, this study recommends a maximum gamma radiation dose of 200 krd.

Based on the achieved results, it is evident that banana fiber nonwoven reinforced composites can be developed using different physical and chemical treatments in the pre- and post-manufacturing stages. The developed materials demonstrate excellent hydrophobicity and comparable mechanical properties, and are able to replace the existing non-biodegradable, carcinogenic and synthetic materials on the market.

## Figures and Tables

**Table 1:** Description of the samples with their designation.

Types	Description	Designation
01	Outer bark nonwoven reinforced epoxy composites	OB/E
02	Middle bark nonwoven reinforced epoxy composites	MB/E
03	Inner bark nonwoven reinforced epoxy composites	IB/E
04	Midrib nonwoven reinforced epoxy composites	MR/E
05	Outer bark nonwoven reinforced polyester composites	OB/P
06	Middle bark nonwoven reinforced polyester composites	MB/P
07	Inner bark nonwoven reinforced polyester composites	IB/P
08	Midrib nonwoven reinforced polyester composites	MR/P

## References

[1] Malviya R.K., Singh R.K., Purohit R., Sinha R. (2019). Natural Fibre Reinforced Composite Materials: Environmentally Better Life Cycle Assessment—A Case Study. Mater. Today.

[2] Yue H., Zheng Y., Zheng P., Guo J., Fernández-Blázquez J.P., Clark J.H., Cui Y. (2020). On the Improvement of Properties of Bioplastic Composites Derived from Wasted Cottonseed Protein by Rational Cross-Linking and Natural Fiber Reinforcement. Green Chem..

[3] Wu Y., Xia C., Cai L., Garcia A.C., Shi S.Q. (2018). Development of Natural Fiber-Reinforced Composite with Comparable Mechanical Properties and Reduced Energy Consumption and Environmental Impacts for Replacing Automotive Glass-Fiber Sheet Molding Compound. J. Clean. Prod..

[4] Kerni L., Singh S., Patnaik A., Kumar N. (2020). A Review on Natural Fiber Reinforced Composites. Mater. Today.

[5] Santhanam V., Dhanaraj R., Chandrasekaran M., Venkateshwaran N., Baskar S. (2020). Experimental Investigation on the Mechanical Properties of Woven Hybrid Fiber Reinforced Epoxy Composite. Mater. Today.

[6] Nayak S.Y., Sultan M.T.H., Shenoy S.B., Kini C.R., Samant R., Shah A.U.M., Amuthakkannan P. (2020). Potential of Natural Fibers in Composites for Ballistic Applications—A Review. J. Nat. Fibers.

[7] Keya K.N., Kona N.A., Koly F.A., Maraz K.M., Islam M.N., Khan R.A. (2019). Natural Fiber Reinforced Polymer Composites: History, Types, Advantages, and Applications. Mater. Eng. Res..

[8] Le Phuong H.A., Ayob N.A.I., Blanford C.F., Rawi N.F.M., Szekely G. (2019). Nonwoven Membrane Supports from Renewable Resources: Bamboo Fiber Reinforced Poly(Lactic Acid) Composites. ACS Sustain. Chem. Eng..

[9] Lotfi A., Li H., Dao D.V., Prusty G. (2021). Natural Fiber–Reinforced Composites: A Review on Material, Manufacturing, and Machinability. J. Thermoplast. Compos. Mater..

[10] Zhang W., Wu J., Gao L., Zhang B., Jiang J., Hu J. (2021). Recyclable, Reprocessable, Self-Adhered and Repairable Carbon Fiber Reinforced Polymers Using Full Biobased Matrices from Camphoric Acid and Epoxidized Soybean Oil. Green Chem..

[11] Pan H., Wang Z., Nie S., Yu L., Chang Y., Liu Z., Xu J., Fu Y. (2021). Novel Green Three-Constituent Natural Deep Eutectic Solvent Enhances Biomass Extraction from Acanthopanax Senticosus and the Extraction Mechanism. ACS Sustain. Chem. Eng..

[12] Voros V., Drioli E., Fonte C., Szekely G. (2019). Process Intensification via Continuous and Simultaneous Isolation of Antioxidants: An Upcycling Approach for Olive Leaf Waste. ACS Sustain. Chem. Eng..

[13] Kenned J.J., Sankaranarayanasamy K., Binoj J.S., Chelliah S.K. (2020). Thermo-Mechanical and Morphological Characterization of Needle Punched Non-Woven Banana Fiber Reinforced Polymer Composites. Compos. Sci. Technol..

[14] Adeniyi A.G., Ighalo J.O., Onifade D.V. (2019). Banana and Plantain Fiber-Reinforced Polymer Composites. J. Polym. Eng..

[15] Balaji A., Purushothaman R., Udhayasankar R., Vijayaraj S., Karthikeyan B. (2020). Study on Mechanical, Thermal and Morphological Properties of Banana Fiber-Reinforced Epoxy Composites. J. Bio. Tribo-Corros..

[16] Srinivasan T., Suresh G., Ramu P., Gokul Ram V., Giresh M., Arjun K. (2020). Effect of Water Absorption of the Mechanical Behavior of Banana Fiber Reinforced IPN Natural Composites. Mater. Today Proc..

[17] Gholampour A., Ozbakkaloglu T. (2020). A Review of Natural Fiber Composites: Properties, Modification and Processing Techniques, Characterization, Applications. J. Mater. Sci..

[18] Komal U.K., Verma V., Ashwani T., Verma N., Singh I. (2020). Effect of Chemical Treatment on Thermal, Mechanical and Degradation Behavior of Banana Fiber Reinforced Polymer Composites. J. Nat. Fibers.

[19] Al-Oqla F.M., Salit M.S. (2017). Materials Selection for Natural Fiber Composites.

[20] Peças P., Carvalho H., Salman H., Leite M. (2018). Natural Fibre Composites and Their Applications: A Review. J. Compos. Sci..

[21] Al-Oqla F.M., Sapuan S.M. (2014). Natural Fiber Reinforced Polymer Composites in Industrial Applications: Feasibility of Date Palm Fibers for Sustainable Automotive Industry. J. Clean. Prod..

[22] Oliveira M.S., Pereira A.C., da Costa Garcia Filho F., da Cruz Demosthenes L.C., Nunes L.F., de Oliveira Braga F., da Luz F.S., Monteiro S.N. (2019). Comparison of Interfacial Adhesion Between Polyester and Epoxy Matrix Composites Reinforced with Fique Natural Fiber. Minerals, Metals and Materials Series.

[23] Sreekumar P.A., Thomas S. (2008). Matrices for natural-fibre reinforced composites. Properties and Performance of Natural-Fibre Composites.

[24] Faruk O., Bledzki A.K., Fink H.P., Sain M. (2014). Progress Report on Natural Fiber Reinforced Composites. Macromol. Mater. Eng..

[25] Binti Mohd Hafidz N.S., Bin Mohamed Rehan M.S., Binti Mokhtar H. (2021). Effect of Alkaline Treatment on Water Absorption and Thickness Swelling of Natural Fibre Reinforced Unsaturated Polyester Composites. Mater. Today Proc..

[26] Mohd Nazarudin Z., Mohd Ariff J., Masitah A.K., Othman N.S., Maizatulnisa O., Syaidatul Hazira M.N., Mohammad Taib M.N.A. (2013). The Effect of Alkaline Treatment on Water Absorption and Tensile Properties of Non-Woven Kenaf Polyester Composite. Adv. Mater. Res..

[27] Manalo A.C., Wani E., Zukarnain N.A., Karunasena W., Lau K.T. (2015). Effects of Alkali Treatment and Elevated Temperature on the Mechanical Properties of Bamboo Fibre-Polyester Composites. Compos. Part B.

[28] Preet Singh J.I., Dhawan V., Singh S., Jangid K. (2017). Study of Effect of Surface Treatment on Mechanical Properties of Natural Fiber Reinforced Composites. Mater. Today.

[29] Wijianto, Ibnu R.M.D., Adityarini H. (2019). Effect of Naoh Concentration Treatment on Tensile Strength, Flexure Strength and Elasticity Modulus of Banana Fiber Reinforced Polyester Resin. Mater. Sci. Forum.

[30] Yan L., Chouw N., Yuan X. (2012). Improving the Mechanical Properties of Natural Fibre Fabric Reinforced Epoxy Composites by Alkali Treatment. J. Reinf. Plast. Compos..

[31] Noura H., Amar B., Hocine D., Rabah Y., Corn S., Roland E.H., Bergeret A. (2018). Effect of Gamma Irradiation Aging on Mechanical and Thermal Properties of Alfa Fiber–Reinforced Polypropylene Composites: Role of Alfa Fiber Surface Treatments. J. Thermoplast. Compos. Mater..

[32] Masudur Rahman A.N.M., Alimuzzaman S., Khan R.A., Hossen J. (2018). Evaluating the Performance of Gamma Irradiated Okra Fiber Reinforced Polypropylene (PP) Composites: Comparative Study with Jute/PP. Fash. Text..

[33] Khan M.A., Khan R.A., Haydaruzzaman, Hossain A., Khan A.H. (2009). Effect of Gamma Radiation on the Physico-Mechanical and Electrical Properties of Jute Fiber-Reinforced Polypropylene Composites. J. Reinf. Plast. Compos..

[34] Haydaruzzaman, Khan R.A., Khan M.A., Khan A.H., Hossain M.A. (2009). Effect of Gamma Radiation on the Performance of Jute Fabrics-Reinforced Polypropylene Composites. Radiat. Phys. Chem..

[35] Martínez-Barrera G., Martínez-López A., Vigueras-Santiago E., Martínez-López M. (2020). Effects of Gamma Radiation on the Physicochemical Properties of Polyester Resin and Its Use in Composite Materials.

[36] Jordan W., Chester P. (2017). Improving the Properties of Banana Fiber Reinforced Polymeric Composites by Treating the Fibers. Procedia Eng..

[37] Mohan T.P., Kanny K. (2019). Compressive Characteristics of Unmodified and Nanoclay Treated Banana Fiber Reinforced Epoxy Composite Cylinders. Compos. Part B.

[38] Sengupta S., Debnath S., Ghosh P., Mustafa I. (2020). Development of Unconventional Fabric from Banana (Musa Acuminata) Fibre for Industrial Uses. J. Nat. Fibers.

[39] Thilagavathi G., Pradeep E., Kannaian T., Sasikala L. (2010). Development of Natural Fiber Nonwovens for Application as Car Interiors for Noise Control. J. Ind. Text..

[40] Manimaran P., Pillai G.P., Vignesh V., Prithiviraj M. (2020). Characterization of Natural Cellulosic Fibers from Nendran Banana Peduncle Plants. Int. J. Biol. Macromol..

[41] Cichosz S., Masek A. (2020). IR Study on Cellulose with the Varied Moisture Contents: Insight into the Supramolecular Structure. Materials.

[42] Alavudeen A., Rajini N., Karthikeyan S., Thiruchitrambalam M., Venkateshwaren N. (2015). Mechanical Properties of Banana/Kenaf Fiber-Reinforced Hybrid Polyester Composites: Effect of Woven Fabric and Random Orientation. Mater. Des..

[43] Mostafa M., Uddin N. (2016). Experimental Analysis of Compressed Earth Block (CEB) with Banana Fibers Resisting Flexural and Compression Forces. Case Stud. Constr. Mater..

[44] Li X., Tabil L.G., Panigrahi S. (2007). Chemical Treatments of Natural Fiber for Use in Natural Fiber-Reinforced Composites: A Review. J. Polym. Environ..

[45] Reddy B.M., Venkata Y., Reddy M., Chandra B., Reddy M. (2018). Effect of Alkali Treatment on Mechanical, Water Absorption and Chemical Resistance Properties of Cordia-Dichotoma Fiber Reinforced Epoxy Composites. Int. J. Appl. Eng. Res..

[46] Bae G.Y., Min B.G., Jeong Y.G., Lee S.C., Jang J.H., Koo G.H. (2009). Superhydrophobicity of Cotton Fabrics Treated with Silica Nanoparticles and Water-Repellent Agent. J. Colloid Interface Sci..

[47] Chowdhury K.P. (2018). Impact of Different Water Repellent Finishes on Cotton Double Jersey Fabrics. J. Text. Sci. Technol..

[48] Yang J., Ching Y.C., Chuah C.H. (2019). Applications of Lignocellulosic Fibers and Lignin in Bioplastics: A Review. Polymers.

[49] Motaleb K.Z.M.A., Al Mizan R., Milašius R. (2020). Development and Characterization of Eco-Sustainable Banana Fiber Nonwoven Material: Surface Treatment, Water Absorbency and Mechanical Properties. Cellulose.

[50] Sivakandhan C., Balaji R., Loganathan G.B., Madan D., Murali G. (2020). Investigation of Mechanical Behaviour on Sponge/Ridge Gourd (Luffa Aegyptiaca) Natural Fiber Using Epoxy and Polyester Resin. Mater. Today.

[51] Rohen L.A., Neves A.C.C., dos Santos J.L., Nascimento L.F.C., Monteiro S.N., de Assis F.S., Simonassi N.T., da Silva L.C. (2018). Comparative Analysis of the Tensile Properties of Polyester and Epoxy Composites Reinforced with Hemp Fibers. Mat. Sci. Forum.

[52] Gnatowski A., Kijo-Kleczkowska A., Gołębski R., Mirek K. (2020). Analysis of Polymeric Materials Properties Changes after Addition of Reinforcing Fibers. Int. J. Numer. Methods Heat Fluid Flow.

[53] EL-Zayat M.M., Abdel-Hakim A., Mohamed M.A. (2019). Effect of Gamma Radiation on the Physico Mechanical Properties of Recycled HDPE/Modified Sugarcane Bagasse Composite. J. Macromol. Sci. Part A Pure Appl. Chem..

